# Effect of Treatment Delay, Stroke Type, and Thrombolysis on the Effect of Glyceryl Trinitrate, a Nitric Oxide Donor, on Outcome after Acute Stroke: A Systematic Review and Meta-Analysis of Individual Patient from Randomised Trials

**DOI:** 10.1155/2016/9706720

**Published:** 2016-04-17

**Authors:** Philip M. Bath, Lisa Woodhouse, Kailash Krishnan, Craig Anderson, Eivind Berge, Gary A. Ford, Thompson G. Robinson, Jeffrey L. Saver, Nikola Sprigg, Joanna M. Wardlaw, Blood pressure in Acute Stroke Collaboration (BASC)

**Affiliations:** ^1^Stroke Trials Unit, Division of Clinical Neuroscience, University of Nottingham, Nottingham NG5 1PB, UK; ^2^Neurological & Mental Health Division, University of Sydney, Sydney, NSW 2000, Australia; ^3^Department of Internal Medicine, Oslo University Hospital, 0424 Oslo, Norway; ^4^Oxford Academic Health Sciences Network, Oxford University Hospitals, NHS Trust, Oxford OX4 4GA, UK; ^5^Department of Cardiovascular Sciences and NIHR Biomedical Research Unit for Cardiovascular Disease, University of Leicester, Leicester LE3 9QP, UK; ^6^Geffen School of Medicine at UCLA, UCLA Comprehensive Stroke Center, Los Angeles, CA 90095, USA; ^7^Division of Neuroimaging Sciences, Centre for Clinical Brain Sciences, University of Edinburgh, Edinburgh EH16 4SB, UK; ^8^University of Nottingham, Nottingham NG5 1PB, UK

## Abstract

*Background.* Nitric oxide (NO) donors are a candidate treatment for acute stroke and two trials have suggested that they might improve outcome if administered within 4–6 hours of stroke onset. We assessed the safety and efficacy of NO donors using individual patient data (IPD) from completed trials.* Methods.* Randomised controlled trials of NO donors in patients with acute or subacute stroke were identified and IPD sought from the trialists. The effect of NO donor versus control on functional outcome was assessed using the modified Rankin scale (mRS) and death, by time to randomisation. Secondary outcomes included measures of disability, mood, and quality of life.* Results.* Five trials (4,197 participants) were identified, all involving glyceryl trinitrate (GTN). Compared with control, GTN lowered blood pressure by 7.4/3.3 mmHg. At day 90, GTN did not alter any clinical measures. However, in 312 patients randomised within 6 hours of stroke onset, GTN was associated with beneficial shifts in the mRS (odds ratio (OR) 0.52, 95% confidence interval (CI) 0.34–0.78) and reduced death (OR 0.32, 95% CI 0.14–0.78).* Conclusions.* NO donors do not alter outcome in patients with recent stroke. However, when administered within 6 hours, NO donors might improve outcomes in both ischaemic and haemorrhagic stroke.

## 1. Introduction

Existing evidence-based treatment options for patients with acute ischaemic stroke may be categorised as those with significant efficacy but limited utility, such as intravenous thrombolysis, thrombectomy, and hemicraniectomy [1–6]; those with limited efficacy but wide utility, for example, aspirin [7]; and stroke unit care with intermediate efficacy and very wide utility [8]. There are no definitive treatments for patients with spontaneous intracerebral haemorrhage (ICH) although very early blood pressure (BP) lowering may be effective and is recommended in guidelines [9, 10]. Hence, there is an urgent need for new interventions that will improve outcome after either ischaemic or haemorrhagic stroke.

Nitric oxide (NO) is a nonorganic gas that has multiple roles in human physiology including vasodilation, BP regulation, antiplatelet and antileucocyte activity, and neurotransmission [11–14]. Circulating NO levels are low in acute stroke [15, 16] and so immediate supplementation might help restore homeostasis through effects on lowering blood pressure, improving cerebral and collateral blood flow, prevention of microthrombosis, reduction of leukocyte adhesion, anti-inflammatory effects, and neuroprotection. NO donors have been studied in animal models of stroke and exhibited time-dependent therapeutic properties [17]. In view of its physiological properties and potential effects in experimental stroke, NO is a candidate treatment for patients with acute stroke [18].

Two NO donors have been assessed in patients with acute or recent stroke. In a small and uncontrolled study, sodium nitroprusside, a spontaneous NO donor, reduced BP and platelet function and did not alter cerebral blood flow (CBF) [19]. However, the NO donor studied most is glyceryl trinitrate (GTN), an organic nitrate licensed for the management of angina. Here, we report a systematic review of the safety and efficacy of NO donors in acute stroke using individual patient data from randomised controlled trials. Since NO donors might be beneficial when administered early, as seen in preclinical studies [17], a small clinical trial [20], and a prespecified subgroup of a large trial [21], we hypothesised that very early administration (defined as randomisation within 6 hours of onset) might be especially effective in improving clinical outcome.

## 2. Methods

### 2.1. Ethics

No research ethics committee approval was needed for this study since anonymised individual patient data came from completed and published trials, each of which had their own national and local approvals and consent.

### 2.2. Selection Criteria and Search Strategy

Completed randomised controlled trials that investigated the effect of a NO donor versus control (placebo or absence of a NO donor) in adult patients with acute or subacute stroke (ischaemic stroke or ICH, within 1 week/168 hours of onset) were sought with searches of electronic databases including the Cochrane Stroke Group Trials Register (searched October 2014), Cochrane Database of Systematic Reviews (CDSR), and the Cochrane Central Register of Controlled Trials (CENTRAL, Cochrane Library Issue 2, 2014), MEDLINE (Ovid) (1966 to May 2014), EMBASE (Ovid) (1974 to May 2014), Science Citation Index (ISI, Web of Science, 1981 to May 2014), and the Stroke Trials Registry (http://www.strokecenter.org/trials/) (May 2014). Separate search strategies were developed for each database (supplemental search criteria). Reference lists in earlier reviews of NO donors and BP lowering [22] and identified trial publications were also checked for additional studies. Whereas duplicate publications were identified, data from the primary report were used. Publications could be in any language.

### 2.3. Outcomes

The primary outcome was dependency at end of follow-up assessed using the 7-level modified Rankin scale (mRS, normal/no symptoms = 0, severe dependency = 5, and dead = 6). Secondary measures during or at the end of randomised treatment included haemodynamics (BP, heart rate); deterioration; recurrence; impairment (Scandinavian Stroke Scale, SSS); headache; symptomatic hypotension; and hypertension of clinical importance. Hospital measures comprised hospital discharge disposition and length of stay. End of follow-up outcomes included disability/activities of daily living (e.g., Barthel index, BI); quality of life (e.g., EuroQoL-5D, EQ-5D; EuroQol-Visual Analogue Scale, EQ-VAS); cognition (e.g., mini-mental state examination, MMSE; telephone interview cognition scale, TICS; semantic fluency/animal naming); mood (e.g., Zung depression scale, ZDS); and place of residence. Safety measures comprised death and serious adverse events (SAEs).

### 2.4. Data

Individual patient data for completed trials were sought from each chief investigator, with data shared electronically (e.g., in Excel, SAS, or SPSS format). Data included information on baseline factors: demographics, vascular risk factors, haemodynamics, stroke type (ischaemic stroke; ICH), stroke severity (e.g., Scandinavian Stroke Scale, SSS), time from onset to randomisation (OTR, as a surrogate for time to treatment), and use of thrombolysis and outcomes. Data from each trial were compared with published results to ensure integrity of the data and analyses.

### 2.5. Trial Quality

Trial quality followed Cochrane collaboration criteria [23] and assessed the following components: method of randomisation; allocation concealment; blinding of treatment administration; blinding of outcome assessment; completeness of outcome data; selective reporting; and any other bias. The assessment for each component in the included study was classified as “low risk,” “high risk,” or “unclear risk” according to the Cochrane Handbook for Systematic Reviews of Interventions [23].

### 2.6. Statistics

Since death is a common outcome after stroke and to avoid missing an effect whereby a treatment might improve both outcome and death, an extreme value was added to the outcomes, as is done routinely for the mRS (death = 6) and EQ-5D (death = 0); the following extreme values were used for death: BI −5, animal naming −1, EQ-VAS −1, TICS-M −1, tMMSE −1, HUS 0, mRS 6, and ZDS 102.5 [21]. The effect of treatment was assessed using binary logistic regression (for binary data such as headache), Cox regression (for time-to-event analyses, e.g., death), ordinal logistic regression (OLR, for ordered categorical data, e.g., mRS), and multiple regression (for continuous or pseudo-continuous data, e.g., SBP, BI). The assumption of proportionality of odds for OLR was tested using the likelihood ratio. The effects of NO donor on subsequent events or outcomes are expressed as odds ratio (OR) or mean difference (MD), with 95% confidence intervals. Statistical models incorporated outcome adjusted for time from onset to randomisation, age, sex, stroke type (ischaemic, haemorrhagic), stroke severity (SSS), stroke syndrome (TACS [24]), and systolic BP. The effect of treatment on the primary outcome (mRS) was assessed in prespecified subgroups in all patients and in those randomised within 6 hours; subgroups were defined for baseline variables: age (<70, ≥70 years), sex, stroke type (IS, ICH), history of hypertension, history of stroke, stroke severity (SSS > 35, ≤35), mean systolic BP (<170, ≥170 mmHg), time from stroke to randomisation (<3, ≥3 hours), treatment with alteplase, and trial. Subgroup analyses were performed by adding an interaction term to an adjusted OLR model. Data are number (%), median (interquartile range), or mean (standard deviation). Analyses were performed using SAS version 9.3; *p* < 0.05 is considered significant.

## 3. Results

### 3.1. Included Trials

Eight studies involving nitric oxide donors in acute stroke were identified. Of these, three were excluded: a completed hospital-based uncontrolled study of sodium nitroprusside [19], an ongoing prehospital ambulance-based phase I uncontrolled study of GTN (http://clinicaltrials.gov/show/NCT01811693), and an ongoing prehospital ambulance-based phase III randomised controlled study of GTN (http://www.isrctn.com/ISRCTN26986053). The five included studies were randomised controlled trials that assessed GTN in patients with acute stroke (see Supplemental Figure I at Supplementary Material available online at http://dx.doi.org/10.1155/2016/9706720), and individual patient data were obtained for each [20, 21, 25–27]. Four trials were small single centre phase II studies [20, 25–27] whilst the largest one, ENOS [21], recruited 4011 patients from 173 sites across 23 countries (Supplemental Table I). One study recruited patients from the community with paramedics leading enrolment, consent, and initial treatment [20]; the other four studies recruited from hospital-based stroke services during the acute and subacute period after stroke [21, 25–27]. When administered, intravenous thrombolysis was given after randomisation to GTN/control in the ambulance-based trial and before randomisation in the large hospital-based trial. One trial was placebo-controlled [25], one was open-label [26], and the other 3 were single blind [20, 21, 27]. All five studies administered GTN as a transdermal patch at 5 mg per day; one trial also tested 10 mg per day in a subgroup of 20 patients (Supplemental Table I) [26]; due to the small number of patients randomised to 10 mg and the finding that it had little extra effect on BP [26], the influence of dose is ignored in the present analysis. In addition to differences in design, the trials differed in respect of patient characteristics including demographics (age, sex distribution), medical history (hypertension, diabetes mellitus), systolic blood pressure, stroke (severity, type), time from onset to randomisation, and use of alteplase (Table 1).

### 3.2. Quality of the Evidence

The overall quality of the included studies was good (Supplemental Table  1). Method of randomisation was clearly reported in each study. Details of BP recording, number of readings, and equipment used were provided. Not all trials contributed to each outcome. Outcome assessment was blinded, as listed in the trial publications (and protocols when available). All participants were accounted for and few patients were lost to follow-up.

### 3.3. Enrolled Patients

Altogether, the trials recruited a total of 4197 participants with 2113 randomised to GTN and 2084 to no GTN (Table 1). The mean (standard deviation) age was 70.4 (12.1) years with 2383 (56.8%) being male. A history of hypertension, stroke, or ischaemic heart disease was present, respectively, in 2700 (64.3%), 623 (15.0%), and 686 (16.5%) of patients. At baseline, 3976 (94.7%) patients had an elevated BP (systolic BP > 140 mmHg) and the mean BP was 167.1 (19.3)/89.5 (13.3) mmHg. 3502 (83.4%) of patients had an ischaemic stroke and 646 (15.4%) an ICH. The mean time from stroke onset to randomisation was 27.2 (16.1) hours, and 312 (7.4%) patients were randomised within 6 hours of ictus. 435 (10.4%) of patients received thrombolytic treatment. The characteristics of patients varied by time to randomisation for several baseline characteristics: sex, history of hypertension and ischaemic heart disease, stroke severity (Scandinavian Stroke Scale), systolic and diastolic blood pressure, heart rate, stroke type (ischaemic, ICH), and treatment with alteplase (Supplemental Table II). In comparison with patients randomised later, those randomised within 6 hours were less likely to have diabetes and more likely to have hypertension (systolic BP > 140 mmHg), presentation with an ICH, and have received rt-PA (if qualifying event was an ischaemic stroke). In patients randomised within 6 hours, baseline characteristics were balanced apart from a history of previous stroke and heart rate, both of which were higher in those randomised to GTN (Supplemental Table II).

### 3.4. Clinical Outcomes

Following first treatment, BP fell by an average of 7.4/3.3 mmHg at 1-2 hours with GTN as compared to no GTN; in contrast, heart rate increased by 1.9 bpm with GTN (Table 2). GTN was associated with increased rates of headache (369/2033, 18.2% versus 171/2026, 8.4%) and clinical hypotension (i.e., hypotension requiring medical intervention, 55/2033, 2.7% versus 15/2026, 0.7%). There were no differences in the rates of death, deterioration, recurrent stroke, clinical hypertension, or serious adverse events by the end of the 7 to 12 days of randomised treatment. Similarly, there were no differences in the rate of death or institutionalisation or length of stay at discharge from hospital (Table 2).

**Figure 1:**
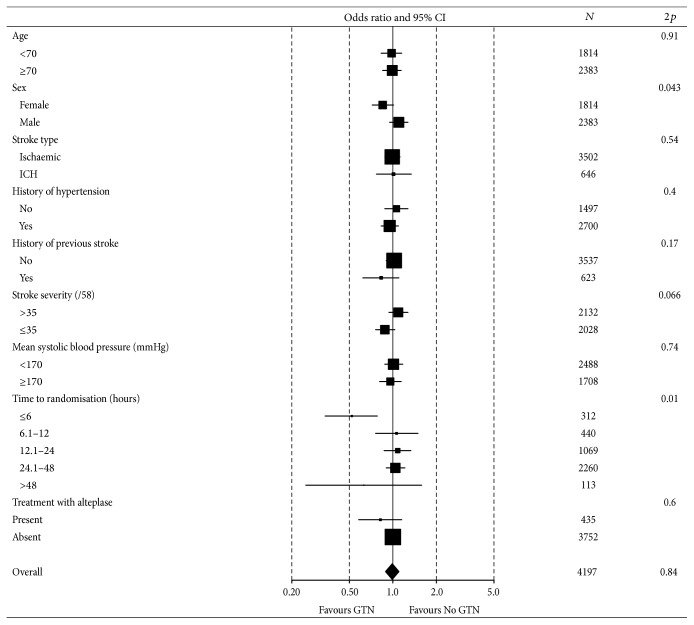
Effect of glyceryl trinitrate versus no glyceryl trinitrate on functional outcome (modified Rankin scale) at 90 days in predefined subgroups of patients. Analyses are adjusted.

No differences were seen between GTN and no GTN for any outcome measure recorded at day 90, including death, dependency (mRS), disability (Barthel index), cognition (MMSE, TICS, and semantic/animal naming), mood (ZDS), or quality of life (EQ-5D as HUS, EQ-VAS). When assessed in prespecified subgroups a time to treatment interaction was present (*p* = 0.01) with patients treated with GTN within 6 hours having a better mRS score (Figure 1). Additionally, an interaction with sex was present (*p* = 0.043) with efficacy only apparent in women. No other subgroup interactions were apparent, including age, vascular risk factors, stroke type, stroke severity, systolic BP, time from onset to randomisation, and use of alteplase.

### 3.5. Outcomes by Time from Stroke to Randomisation

**Figure 2:**
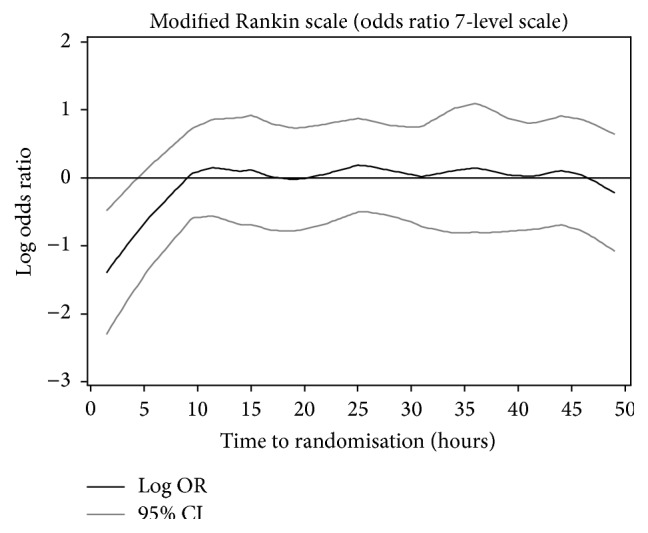
Effect of glyceryl trinitrate versus no glyceryl trinitrate on functional outcome (modified Rankin scale) at 90 days by time to randomisation.

**Figure 3:**
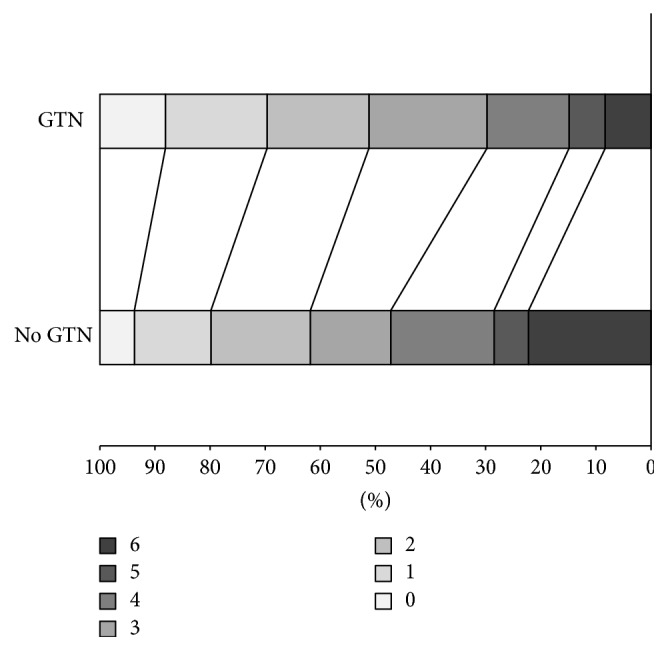
Distribution in modified Rankin scale scores at day 90 for glyceryl trinitrate versus no glyceryl trinitrate in 312 patients with any stroke and randomised within 6 hours of stroke onset. Common odds ratio 0.52 (95% confidence intervals 0.34–0.78; *p* = 0.002).

Analysis of mRS by time to randomisation showed no effect beyond 5–10 hours and out to 50 hours, but apparent efficacy (shift in mRS) with earlier treatment (Figure 2). 312 patients were randomised within 6 hours of stroke onset, these coming from two of the five trials (ENOS, RIGHT) [20, 21]. The earliest time from stroke to randomisation was 7 minutes. At 90 days, patients randomised to GTN within 6 hours had significant reductions in death, dependency (lower mRS scores, Figure 3), disability (higher Barthel index scores), and mood disturbance (lower ZDS scores) and higher cognition scores (higher tMMSE and TICS scores) (Table 2). Quality of life, semantic fluency (animal naming), death or institutionalisation, and rate of serious adverse events did not differ between the treatment groups.

No interactions between treatment and mRS in prespecified subgroups were present (Supplemental Figure II). A trend to more benefit in men than women (and the converse across all studies and times to recruitment) is likely to reflect chance. Significant shifts to less death or dependency were seen with GTN for both haemorrhagic and ischaemic stroke (Supplemental Figures III, IV). In patients with an ischaemic stroke, a significant shift to less death or dependency was seen for patients who received both GTN and thrombolysis (Supplemental Figure V). However, benefit was not seen in those with an ischaemic stroke who did not receive thrombolysis (Supplemental Figure VI); a trend to benefit from GTN was seen in a* post hoc* unadjusted comparison using a Mann-Whitney *U* test (*p* = 0.092).

**Figure 4:**
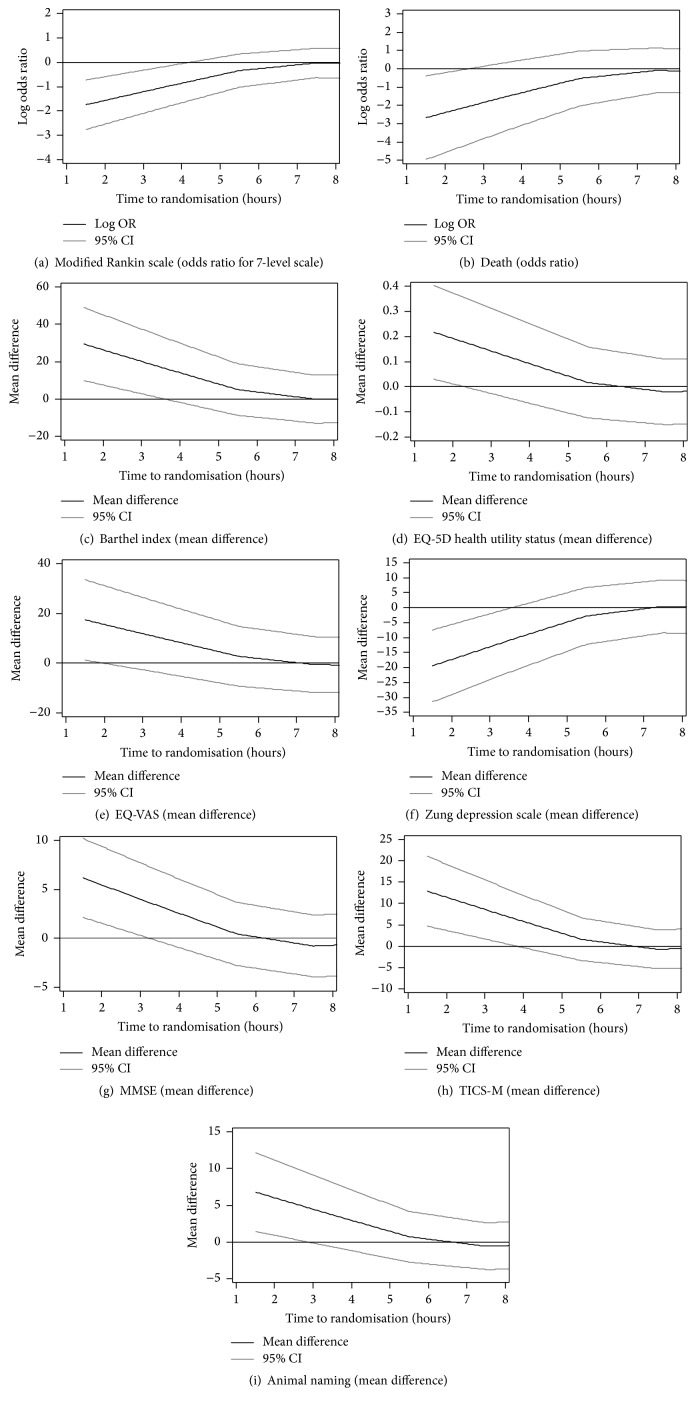
Effect of GTN on outcome by time to enrolment within 8 hours for (a) modified Rankin scale; (b) death; (c) Barthel index; (d) Euro-Qol 5D (EQ-5D) health utility status; (e) Euro-QoL Visual Analogue Scale (EQ-VAS); (f) Zung depression scale; (g) telephone mini-mental state examination; (h) telephone interview of cognition scale, modified (TICS-M); and (i) animal naming.

There was no overall evidence of benefit or harm, beyond 6 hours with GTN versus no GTN. Lower cognition scores were apparent for patients randomised to GTN beyond 48 hours (Table 2). When considering the continuous relationship between outcome and time to randomisation, time-dependent effects of GTN were apparent in patients randomised within 6 hours of onset for mRS, BI, EQ-5D, EW-VAS, ZDS, and death (Figure 4).

## 4. Discussion

NO donors are a candidate treatment for acute stroke [18]. Five randomised controlled trials have assessed GTN, an organic nitrate, in 4197 patients with acute ischaemic stroke or ICH [20, 21, 25–27]. Overall, GTN had no effects on functional outcome, disability, cognition, mood, quality of life, or death. However, in comparison with no GTN, patients randomised to GTN within 6 hours of stroke onset, a prespecified subgroup [21], had a better outcome at day 90, assessed using multiple markers of physical and mental functional performance. Importantly, GTN appeared to improve outcome in this subgroup in both ischaemic stroke and ICH and in patients who received intravenous thrombolysis. Further, very early treatment appeared to be safe in patients enrolled without a stroke.

Although the apparent benefit seen in patients randomised within 6 hours could reflect chance, several points suggest the finding may be real. Firstly, beneficial effects of very early administration of GTN were seen independently in two trial datasets: RIGHT (median time to treatment 55 minutes) and the ENOS subgroup treated within 6 hours (median time 258 minutes) had odds ratios for mRS of 0.08 (95% CI 0.02–0.41) and 0.57 (95% CI 0.37–0.89), respectively [20, 21]. Secondly, the effect was apparent in a study population of more than 300 patients, a size similar to each of the positive components of the NINDS trial of alteplase [28] and in a recent trial of mechanical thrombectomy [5]. Thirdly, a time-dependent effect within the 6-hour time frame was apparent, with the most potent effect seen in patients treated very early during this period. Fourthly, a positive effect was seen for multiple different outcomes covering death, dependency, disability, cognition, mood, and quality of life. Fifthly, the effect of treatment when given early was present across all prespecified subgroups and was independent of stroke type and severity and baseline BP. And last, the time-dependent neuroprotective effect was also reported in a meta-analysis of preclinical stroke studies of NO donors which showed that studies treating within 60 minutes of initiation of ischaemia were positive whereas those with a longer time window (up to 48 hours) were neutral [17]. Importantly, those variables that differed between treatment groups in patients randomised within 6 hours (history of previous stroke and heart rate, both of which were higher with GTN) are unlikely to have explained the differences in outcome seen in favour of GTN.

If hyperacute administration of GTN is beneficial after stroke, then a number of potential mechanisms can be postulated. Taken together, these actions may “buy time” and protect the brain and prepare patients with ischaemic stroke for thrombolysis. Circulating NO levels are low in acute stroke [15, 16], perhaps due to local failure of production by damaged endothelium; hence, immediate supplementation might help restore this focal deficiency. A mechanism of potential relevance to both ischaemic and haemorrhagic stroke is that NO/GTN lowers BP in acute/subacute stroke [20, 21, 25–27] and so may move hypertensive patients down the “J-shaped” epidemiological curve relating BP and poor functional outcome [29] towards its nadir. Lowering BP might reduce early recurrence after ischaemic stroke and haematoma expansion in ICH [9, 30]. A number of additional mechanisms apply to ischaemic stroke specifically. Firstly, NO donors are neuroprotective in preclinical ischaemic stroke [17], especially if given ultra-early. Secondly, NO dilates cerebral arteries (e.g., middle cerebral) so it could increase perilesional perfusion (via the “front door”) but without inducing steal to other brain areas, as seen in the GTN-3 pilot trial [27]. Thirdly, NO is a powerful dilator of pial arteries (as shown experimentally [31]) and so might increase tissue perfusion via the “backdoor” collateral system. Fourthly, NO donors have anti-inflammatory effects, for example, through reducing leukocyte adhesion. Fifthly, NO donors can reduce thrombosis, as in microvessels. And last, in addition to increases in blood levels of NO, other potentially beneficial biochemical changes may occur, as seen experimentally and including increases in endothelial nitric oxide synthase (NOS) and cyclic guanosine monophosphate and reductions in neuronal NOS, nitrotyrosine, and adhesion molecules [32–35].

Separately, GTN may “prime” patients for alteplase by lowering their BP to below the licensed maximum of systolic BP of 185 mmHg, thereby allowing more to be treated, and earlier, as hinted at in the RIGHT pilot trial [20]. Additionally, GTN-induced cerebral arterial vasodilation may increase access of alteplase to the obstructing clot and therefore increase the effectiveness of thrombolysis. Hence, the effect of GTN might be additive with alteplase, as suggested here in patients who received both drugs. All of these potential mechanisms are likely to be time-dependent thereby explaining why no positive findings were seen beyond 6 hours. And those mechanisms that improve blood flow and perfusion mimic the time-dependent benefits seen with intravenous thrombolysis and mechanical thrombectomy, both of which improve outcome if delivered within 4.5 or 8 hours, respectively, of stroke onset [1–5].

This study has several strengths. Firstly, it uses individual patient data from all identified completed controlled trials of NO donors. Individual patient data meta-analyses are considered the “gold standard” [36] since they allow for covariate adjusted analyses (and so can adjust for any nonmajor imbalances at baseline) and facilitate analyses within subgroups; the present meta-analysis benefits in both respects. Secondly, it includes a large cohort of more than 4000 patients (comparable in size to a metaregression analysis of the effects of thrombolysis assessed by time to randomisation [37]). And last, it assesses safety and efficacy across a number of physical and mental outcome domains; the results are similar across all the studies outcome domains thereby exhibiting internal consistency.

Several limitations are also apparent. First, all the data relate to GTN and the results and conclusions may not apply to other nitric oxide donors. Secondly, the majority of data come from the ENOS trial (95.6% of all patients and 86.9% of those randomised within 6 hours of onset) and therefore its results dominate the analyses. Nevertheless, addition of the four pilot trials extends the time windows examined in the analyses; RIGHT tested ultra-acute prehospital treatment (<4 hours with median time 55 minutes [20]) whilst the three earlier pilot studies tested times beyond 48 hours. Additionally, patient characteristics differed between the trials and so improved external validity. Thirdly, one of the trials was open-label [26] and three were single blind [20, 21, 27]. rather than double-masked (reflecting the lack of availability from the late 1990s of placebo GTN patches from commercial sources); although each trial used one or more independent assessors blinded to treatment to record the clinical outcomes, the potential for observer bias cannot be ruled out. Additionally, GTN causes headache and this may have unblinded some patients to their treatment assignment. Further, treatment of headache might impact on outcome although there no benefit was seen in a trial of paracetamol [38]. Fourthly, the data all come from one research group and it is important that other research groups study the role of GTN in very early stroke. Last, a relatively small number of patients were treated within 6 hours of stroke onset and these come from just two [20, 21] of the five trials. The results in this prespecified subgroup must be considered provisional and support the need for one or more large multicentre trials recruiting patients in the ultra-acute period after stroke. The RIGHT-2 trial is testing GTN for this indication in 850 patients recruited by paramedics in the prehospital setting (http://right-2.ac.uk/) and other prehospital trials are planned. Importantly, treatment before hospital admission has the potential for modifying in-hospital diagnosis (e.g., converting ischaemic stroke into TIA) and severity, as seen potentially in a trial of ambulance-based preconditioning [39]. Similarly, prehospital treatment may modify the use and need for hospital-based interventions. Whilst the RIGHT trial showed a trend to more and earlier use of thrombolysis [20], reduced neurovascular damage related to treatment with GTN might reduce the need for other interventions such as intra-arterial interventions or therapy; a hint of this was seen in ENOS-early where there was a trend for less physiotherapy in patients randomised to GTN [40].

## 5. Conclusions

In summary, NO donors in general and GTN specifically do not improve outcome after acute and subacute stroke. These results are similar to those seen for nitrates in acute myocardial infarction [41, 42]. Nevertheless, the positive finding in the prespecified subgroup of patients randomised within 6 hours deserves further study, especially since GTN is readily available, inexpensive, and easy to administer and can be administered in the prehospital setting prior to brain scanning.

## Supplementary Material

The supplementary material contains information on the study investigators and includes further data from this systematic review. Supplemental Table 1 contains the characteristics of the trials which were included in this systematic review. Supplemental Table 2 contains the baseline characteristics of enrolled patients from all five studies by time to randomisation. Supplemental Figure 1 contains a flow diagram of the search for eligible trials. Supplemental Figure 2 contains a forest plot of a subgroup analysis of the modified Rankin scale (mRS) at 90 days, for patients randomised within 6 hours of stroke onset. Supplemental Figure 3 contains a shift diagram on the mRS at day 90 in patients with intracerebral haemorrhage who were randomised within 6 hours of stroke onset. Supplemental Figure 4 contains a shift diagram of the mRS at day 90 in patients with ischaemic stroke who were randomised within 6 hours of stroke onset. Supplemental Figure 5 contains a shift diagram of the mRS at 90 days in patients with ischaemic stroke who received alteplase and who were randomised within 6 hours of stroke onset. Supplemental Figure 6 contains a shift diagram of the mRS at 90 days in patients with ischaemic stroke who did not receive alteplase and who were randomised within 6 hours of stroke onset.

## Figures and Tables

**Table 1:** Baseline characteristics of enrolled patients by GTN versus no GTN and by trial. Data from the ENOS trial are divided by time to randomisation (≤6 hours, 6–48 hours). Data are number (%), median (interquartile range), or mean (standard deviation).

Trial	All	GTN	No GTN	GTN-1 [25]	GTN-2 [26]	GTN-3 [27]	RIGHT [20]	ENOS-early [21]	ENOS-rest [21]	2*p*
Number	4197	2113	2084	37	90	18	41	273	3738	
Age (yrs)	70.4 (12.1)	70.4 (12.1)	70.4 (12.2)	73.7 (9.1)	71.8 (11.8)	69.4 (7.4)	76.3 (11)	69.9 (12.7)	70.3 (12.1)	0.012
Male (%)	2383 (56.8)	1198 (56.7)	1185 (56.9)	18 (48.6)	41 (45.6)	5 (27.8)	22 (53.7)	154 (56.4)	2143 (57.3)	0.029
Stroke (%)	623 (15.0)	336 (16.0)	287 (13.9)	NC	15 (16.7)	3 (16.7)	11 (26.8)	39 (14.3)	555 (14.8)	0.30
HT (%)	2700 (64.3)	1338 (63.3)	1362 (65.4)	23 (62.2)	36 (40)	7 (38.9)	27 (65.9)	168 (61.5)	2439 (65.2)	<0.001
DM (%)	715 (17.2)	354 (16.9)	361 (17.5)	NC	12 (13.3)	0 (0.0)	4 (9.8)	37 (13.6)	662 (17.7)	0.049
IHD (%)	686 (16.5)	353 (16.8)	333 (16.1)	NC	13 (14.4)	2 (11.1)	2 (4.9)	34 (12.5)	635 (17)	0.062
AF (%)	597 (14.4)	318 (15.2)	279 (13.5)	NC	18 (20)	0 (0.0)	8 (19.5)	38 (13.9)	533 (14.3)	0.18
SBP (mmHg)	167.1 (19.3)	167.1 (19.2)	167 (19.4)	161.9 (21.5)	159.2 (27)	163.3 (16.5)	172.5 (28.2)	166.7 (18.2)	167.3 (19)	<0.001
Minimum	101	101	110	126	101	137	137	132	110	
Maximum	234	234	233	219	224	191	234	223	233	
>140 (%)	3976 (94.7)	2000 (94.7)	1976 (94.8)	32 (86.5)	62 (68.9)	16 (88.9)	40 (97.6)	262 (96)	3564 (95.3)	<0.001
DBP (mmHg)	89.5 (13.3)	89.7 (13.3)	89.3 (13.4)	91.6 (15.7)	86.8 (16.3)	90.4 (14.9)	92.1 (19.9)	90.8 (13.3)	89.4 (13.1)	0.11
HR (bpm)	77.5 (14.8)	77.8 (14.9)	77.3 (14.6)	77.3 (15.7)	78 (15.2)	68.2 (14)	82.5 (18)	77.5 (14.9)	77.5 (14.7)	0.036
SSS (/58)	33.7 (13.2)	34 (13.1)	33.5 (13.3)	NC	32.6 (11.7)	42.1 (10.5)	35.7 (17)^†^	32.1 (11.9)	33.8 (13.3)	0.010
IS (%)	3502 (83.4)	1759 (83.2)	1743 (83.6)	33 (89.2)	84 (93.3)	16 (88.9)	27 (65.9)	208 (76.2)	3134 (83.8)	<0.001
ICH (%)	646 (15.4)	323 (15.3)	323 (15.5)	4 (10.8)	5 (5.6)	2 (11.1)	6 (14.6)	61 (22.3)	568 (15.2)	0.003
OTR (hours)	27.2 (16.1)	27.1 (16.4)	27.2 (15.8)	99.2 (38)	55.1 (18.9)	72.2 (26.2)	1.6 (1.8)	4.3 (1.2)	27.6 (11.9)	<0.001
<6 hours	312 (7.4%)	168 (8)	144 (6.9)	0 (0.0)	0 (0.0)	0 (0.0)	39 (95.1)	273 (100)	0 (0.0)	<0.001
rt-PA (%)^‡^	435 (10.4)	211 (10)	224 (10.7)	0 (0.0)	0 (0.0)	0 (0.0)	10 (24.4)	93 (34.1)	332 (8.9)	<0.001

^†^Stroke severity measured after randomisation at hospital admission.

^‡^Percentage includes IS and ICH patients.

AF: atrial fibrillation; bpm: beats per minute; DBP: diastolic blood pressure; DM: diabetes mellitus; GTN: glyceryl trinitrate; HR: heart rate; HT: hypertension; ICH: intracerebral haemorrhage; IHD: ischaemic heart disease; IS: ischaemic stroke; OTR: onset time to randomisation; rt-PA: recombinant tissue-plasminogen activator/alteplase; SBP: systolic blood pressure; SSS: Scandinavian Stroke Scale.

**Table 2:** Outcomes by time from stroke onset to randomisation. Data are number (%), mean difference, or odds ratio with 95% confidence intervals. Comparisons by binary logistic regression, ordinal logistic regression, or linear regression, with adjustment for trial. Results in bold are statistically significant, *p* < 0.05.

OTR (hours)	All	≤6	6.1–12	12.1–24	24.1–48	>48
Patients (*N*)	4197	312 (7.43)	440 (10.48)	1069 (25.47)	2260 (53.85)	113 (2.69)
*After first patch*						
SBP (mmHg)	**−7.4 (−8.8, −6.0)**	**−9.4 (−14.3, −4.5)**	**−8.3 (−12.7, −3.9)**	**−5.3 (−8.1, −2.5)**	**−7.5 (−9.4, −5.6)**	**−12.3 (−21.0, −3.7)**
DBP (mmHg)	−**3.3 (−4.2, −2.5)**	−2.4 (−5.7, 0.9)	**−4.5 (−7.4, −1.7)**	**−3.3 (−5.0, −1.6)**	**−3.3 (−4.6, −2.1)**	−0.9 (−5.9, 4.1)
HR (bpm)	**1.9 (1.0, 2.8)**	1.9 (−1.7, 5.6)	−0.1 (−3.1, 3.1)	1.7 (−0.1, 3.5)	**2.1 (0.9, 3.4)**	**4.8 (0.1, 9.5)**
*Treatment end*						
Patients (%)	4188 (99.8)	309 (99.0)	440 (100)	1067 (99.8)	2256 (99.8)	113 (100)
Death	1.15 (0.79, 1.68)	0.94 (0.23, 3.81)	1.52 (0.42, 5.48)	0.89 (0.47, 1.69)	1.37 (0.77, 2.42)	1.00 (0, ∞)
Deterioration	1.29 (1.00, 1.66)	0.58 (0.28, 1.23)	1.94 (0.84, 4.48)	1.22 (0.76, 1.97)	1.45 (1.00, 2.10)	0.86 (0.06, 12.67)
Recurrence	1.39 (0.88, 2.18)	0.46 (0.14, 1.54)	2.15 (0.65, 7.17)	2.24 (0.95, 5.28)	1.04 (0.48, 2.26)	1.77 (0, ∞)
SSS/58	0.33 (−0.26, 0.91)	2.33 (−0.42, 5.09)	−0.19 (−2.20, 1.81)	0.01 (−1.18, 1.20)	0.14 (−0.59, 0.87)	0.84 (−2.08, 3.76)
Headache	**2.42 (1.99, 2.95)**	**2.16 (1.04, 4.48)**	**2.37 (1.29, 4.36)**	**2.55 (1.76, 3.69)**	**2.41 (1.84, 3.16)**	NC
Hypotension	**3.66 (2.06, 6.51)**	2.67 (0.65, 11.01)	**9.64 (1.11, 83.62)**	**14.85 (1.95, 113.18)**	**2.60 (1.24, 5.45)**	NC
Hypertension	0.87 (0.68, 1.10)	0.71 (0.32, 1.60)	1.88 (0.89, 3.98)	0.92 (0.56, 1.50)	0.74 (0.53, 1.04)	0 (0, ∞)
SAEs	1.07 (0.88, 1.29)	0.81 (0.42, 1.55)	**2.04 (1.14, 3.62)**	1.04 (0.74, 1.48)	0.98 (0.74, 1.28)	NC
*End-of-hospital*						
Patients (%)	4173 (99.4)	307 (98.4)	439 (99.7)	1066 (99.7)	2245 (99.3)	113 (100)
Death	1.04 (0.81, 1.32)	0.45 (0.17, 1.18)	1.82 (0.86, 3.81)	1.19 (0.74, 1.91)	0.97 (0.69, 1.37)	0 (0, 0)
Death/institution	0.91 (0.79, 1.04)	0.79 (0.47, 1.33)	1.14 (0.73, 1.77)	0.95 (0.72, 1.25)	0.91 (0.75, 1.10)	0.60 (0.17, 2.14)
LoS (days)	0.07 (−1.3, 1.44)	0.02 (−4.59, 4.63)	−2.54 (−5.87, 0.78)	−0.48 (−3.14, 2.17)	1.16 (−0.73, 3.05)	−1.97 (−20.97, 17.04)
*Day 90*						
Patients (%)	4197 (100)	312 (100)	440 (100)	1069 (100)	2260 (100)	113 (100)
Death	0.87 (0.71, 1.07)	**0.32 (0.14, 0.78)**	1.20 (0.68, 2.11)	1.00 (0.67, 1.50)	0.86 (0.64, 1.14)	0 (0, 0)
mRS	0.99 (0.89, 1.10)	**0.52 (0.34, 0.78)**	1.06 (0.76, 1.49)	1.08 (0.87, 1.33)	1.04 (0.90, 1.21)	0.63 (0.25, 1.58)
BI	1.73 (−0.08, 3.55)	**9.64 (3.19, 16.09)**	0.27 (−5.92, 6.46)	0.03 (−3.57, 3.64)	1.31 (−1.11, 3.72)	3.09 (−6.69, 12.87)
tMMSE	0.34 (−0.16, 0.84)	**2.09 (0.65, 3.54)**	−0.25 (−1.83, 1.33)	−0.18 (−1.10, 0.75)	0.41 (−0.30, 1.11)	−4.49 (−7.37, −1.61))
TICS	0.16 (−0.55, 0.88)	**3.56 (1.20, 5.91)**	0.09 (−2.16, 2.34)	−0.79 (−2.12, 0.54)	0.19 (−0.80, 1.18)	**−9.63 (−12.51, −6.74)**
Animal naming	−0.06 (−0.60, 0.47)	1.63 (−0.13, 3.39)	−0.02 (−1.71, 1.67)	−0.12 (−1.15, 0.90)	−0.25 (−0.98, 0.48)	**−4.68 (−7.3, −2.05)**
ZDS	−0.38 (−1.80, 1.04)	**−8.34 (−13.32, −3.36)**	0.59 (−3.99, 5.16)	1.63 (−1.14, 4.39)	−0.31 (−2.21, 1.58)	4.68 (−11.16, 20.52)
HUS	0 (−0.02, 0.02)	0.05 (−0.02, 0.13)	−0.01 (−0.08, 0.05)	−0.02 (−0.06, 0.02)	0.01 (−0.02, 0.03)	0.06 (−0.1, 0.22)
EQ-VAS	0.69 (−1.06, 2.43)	5.97 (−0.30, 12.24)	−1.16 (−6.91, 4.60)	−0.84 (−4.27, 2.60)	0.55 (−1.79, 2.90)	7.37 (−2.85, 17.59)
Death/institution	0.89 (0.77, 1.03)	0.63 (0.35, 1.11)	1.06 (0.68, 1.67)	0.90 (0.67, 1.21)	0.92 (0.75, 1.12)	0 (0, ∞)
SAEs	1.07 (0.92, 1.25)	0.58 (0.34, 1.01)	1.38 (0.88, 2.17)	1.04 (0.78, 1.39)	1.12 (0.91, 1.38)	0.25 (0, 22.31)

∞ Value tends towards infinity.

Values for death: BI −5, animal naming −1, EQ-VAS −1, TICS-M −1, tMMSE −1, HUS 0, mRS 6, and ZDS 102.5.

BI: Barthel index; bpm: beats per minute; DBP: diastolic blood pressure; EQ: EuroQol; EQ-VAS: EuroQol Visual Analogue Scale; HR: heart rate; HUS: health utility status (derived from EQ-5D); LoS: length of stay; mRS: modified Rankin scale; NC: not collected; OTR: onset to randomisation; SAE: serious adverse event; SBP: systolic blood pressure; SSS: Scandinavian Stroke Scale; TICS: telephone interview cognition scale; tMMSE: telephone mini-mental state examination; ZDS: Zung depression scale.
