# Supplementary material for: Effect of Treatment Delay, Stroke Type, and Thrombolysis on the Effect of Glyceryl Trinitrate, a Nitric Oxide Donor, on Outcome after Acute Stroke: A Systematic Review and Meta-Analysis of Individual Patient from Randomised Trials
Source: Stroke Res Treat. 2016 Apr 17;2016:9706720. doi: 10.1155/2016/9706720 (PMC4852111; doi:10.1155/2016/9706720)
Supplement: Supplementary file 1 — The supplementary material contains information on the study investigators and includes further data from this systematic review. Supplemental Table 1 contains the characteristics of the trials which were included in this systematic review. Supplemental Table 2 contains the baseline characteristics of enrolled patients from all five studies by time to randomisation. Supplemental Figure 1 contains a flow diagram of the search for eligible trials. Supplemental Figure 2 contains a forest plot of a subgroup analysis of the modified Rankin scale (mRS) at 90 days, for patients randomised within 6 hours of stroke onset. Supplemental Figure 3 contains a shift diagram on the mRS at day 90 in patients with intracerebral haemorrhage who were randomised within 6 hours of stroke onset. Supplemental Figure 4 contains a shift diagram of the mRS at day 90 in patients with ischaemic stroke who were randomised within 6 hours of stroke onset. Supplemental Figure 5 contains a shift diagram of the mRS at 90 days in patients with ischaemic stroke who received alteplase and who were randomised within 6 hours of stroke onset. Supplemental Figure 6 contains a shift diagram of the mRS at 90 days in patients with ischaemic stroke who did not receive alteplase and who were randomised within 6 hours of stroke onset. [file 9706720.f1.docx]

**SUPPLEMENTAL MATERIAL**

**TABLE OF CONTENTS**

Page

Title 1

Study Investigators 1

Tables 3

Figures 5

References 11

Search criteria 12

**TITLE**

Effect of treatment delay, stroke type, and thrombolysis on the effect of glyceryl trinitrate, a nitric oxide donor, on outcome after acute stroke: a systematic review and meta-analysis of individual patient from randomised trials

**STUDY INVESTIGATORS**

Blood pressure in Acute Stroke Collaboration (BASC) *

**Writing Committee**

Philip M Bath, Lisa Woodhouse, Kailash Krishnan, Craig Anderson, Eivind Berge, Gary A Ford, Tom Robinson, Jeffrey L Saver, Nikola Sprigg, Joanna M Wardlaw

**BASC Collaborators**

ATACH-2: Adnan Qureshi

CATIS: Jiang He, Yonghong Zhang

CHHIPS: John Potter

COSSACS: Tom Robinson

ENCHANTED: Craig Anderson

ENOS: Philip Bath, Niki Sprigg, Joanna Wardlaw

FAST-Mag: Jeff Saver, Nerses Sanossian

GTN-1/2/3, RIGHT: Philip Bath

ICH ADAPT: Ken Butcher

IMAGES: Kennedy R Lees, Keith W Muir

INTERACT: Craig Anderson

INTERACT-2: Craig Anderson, Histomi Arima, Emma Heeley

PILFAST: Gary Ford

SCAST: Eivind Berge, Else Sandset

STAR: Niki Sprigg

**Contributors**

All authors contributed to the interpretation of the results and writing of this report. PMB wrote the first draft of this report. LW analysed trial data and commented on a draft of this report. Members of the writing committee commented on the draft of this report. All members of the writing committee have seen and approved the final version of this report.

**Funding**

This research received no specific grant from any funding agency in the public, industry or not-for-profit sectors. The included trials were funded by: GTN-1: British Heart Foundation and South Thames NHS Executive; GTN-2: no specific funding; GTN-3: Hypertension Trust; ENOS: BUPA Foundation and Medical Research Council; RIGHT: Nottingham University Hospitals NHS Trust. JMW was supported, in part, by the Scottish Funding Council through the SINAPSE Collaboration ([www.sinapse.ac.uk/](http://www.sinapse.ac.uk/)). PMB is Stroke Association Professor of Stroke Medicine.

**Conflicts of Interest**

PB was Chief Investigator of all the included trials.

**Supplemental Table I. Characteristics of included trials.**

|  | GTN-1 ^1^ | GTN-2 ^2^ | GTN-3 ^3^ | RIGHT ^4-6^ | ENOS ^7-10^ |
| --- | --- | --- | --- | --- | --- |
| Registration | N/A | N/A | N/A | ISRCTN66434824 | ISRCTN99414122 |
| Setting | Hospital | Hospital | Hospital | Pre-hospital/ambulance | Hospital |
| Time window (hr) | <120 hours | <72 hours | <120 hours | <4 hours | <48 hours |
| Stroke type | IS/ICH | IS/ICH | IS/ICH | IS/ICH | IS/ICH |
| Systolic blood pressure, range (mmHg) | - | 100-230 | 140-220 | >140 | 140-220 |
| Treatment blinding | Double-blind | Open-label | Single-blind | Single-blind | Single-blind |
| GTN daily dose (mg) | 5 | 5/10 | 5 | 5 | 5 |
| Length of treatment (days) | 12 | 10 | 7 | 7 | 7 |
| Thrombolysis | N/A | N/A | N/A | After randomised treatment | Before randomised treatment |
| Outcome blinded | Yes | Yes | Yes | Yes | Yes |
| Sample size |  |  |  |  |  |
| Intended | 38 | 90 | 18 | 80 | > 3500 |
| Achieved | 37 | 90 | 18 | 41 | 4011 |
| Trial quality ^11^ |  |  |  |  |  |
| Random sequence generation | Low risk [1] | Low risk [1] | Low risk [1] | Low risk [2] | Low risk [1] |
| Allocation concealment | Low risk | Unclear risk | Low risk | Low risk | Low risk |
| Blinding (performance/detection bias) | Low risk | Unclear risk | Low risk | Unclear risk | Unclear risk |
| Blinding – participants, personnel (performance bias) | Low risk | Unclear risk | Low risk | Unclear risk | Unclear risk |
| Blinding - outcome assessment (detection bias) | Low risk | Low risk | Low risk | Low risk | Low risk |
| Incomplete outcome data (attrition bias) | Low risk | Low risk | Low risk | Low risk | Low risk |
| Selective reporting (reporting bias) | Low risk | Low risk | Low risk | Low risk | Low risk |
| Other bias | Low risk | Low risk | Low risk | Low risk | Low risk |

ICH: intracerebral haemorrhage; IS: ischaemic stroke; N/A: not applicable

[1] Randomisation included minimisation. [2] Simple randomisation.

**Supplemental Table II**. Baseline characteristics of enrolled patients by time to randomisation (hours).

| Time (hours) | All |  | <=6 |  | <=6 | 6.1-12 | 12.1-24 | 24.1-48 | >48 | 2p |
| --- | --- | --- | --- | --- | --- | --- | --- | --- | --- | --- |
|  |  | GTN | No GTN | 2p |  |  |  |  |  |  |
| Number (%) | 4197 | 168 | 144 |  | 312 (7.4) | 440 (10.5) | 1069 (25.5) | 2260 (53.8) | 113 (2.7) |  |
| Age (yrs) | 70.4 (12.1) | 69.8 (11.8) | 71.6 (13.5) | 0.22 | 70.6 (12.6) | 69.4 (12.1) | 70.7 (12.2) | 70.4 (12.1) | 72.0 (10.9) | 0.21 |
| Male (%) | 2383 (56.8) | 89 (53) | 86 (59.7) | 0.23 | 175 (56.1) | 261 (59.3) | 634 (59.3) | 1263 (55.9) | 49 (43.4) | 0.011 |
| Stroke (%) | 623 (15.0) | 35 (20.8) | 15 (10.4) | 0.012 | 50 (16.0) | 73 (16.6) | 144 (13.5) | 342 (15.2) | 14 (17.5) | 0.48 |
| Hypertension (%) | 2700 (64.3) | 110 (65.5) | 84 (58.3) | 0.19 | 194 (62.2) | 294 (66.8) | 693 (64.8) | 1469 (65.0) | 50 (44.2) | < 0.001 |
| DM (%) | 715 (17.2) | 24 (14.3) | 17 (11.8) | 0.52 | 41 (13.1) | 75 (17.0) | 183 (17.1) | 402 (17.8) | 13 (16.3) | 0.37 |
| IHD (%) | 686 (16.5) | 22 (13.3) | 14 (9.9) | 0.36 | 36 (11.8) | 88 (20.4) | 193 (18.4) | 359 (16.3) | 9 (11.5) | 0.01 |
| AF (%) | 597 (14.4) | 24 (14.3) | 21 (14.6) | 0.94 | 45 (14.4) | 64 (14.5) | 166 (15.5) | 313 (13.9) | 7 (8.8) | 0.44 |
| SSS (/58) | 33.7 (13.2) | 33.5 (12) | 31.3 (13.3) | 0.14 | 32.5 (12.6) | 32.8 (13.0) | 33.2 (13.4) | 34.3 (13.2) | 35.9 (12.7) | 0.012 |
| SBP (mmHg) | 167.1 (19.3) | 168 (19.8) | 166.8 (19.7) | 0.58 | 167.4 (19.7) | 169.8 (19.8) | 167.3 (18.9) | 166.7 (19.2) | 161 (21.5) | < 0.001 |
| Minimum | 101 | 137 | 132 |  | 132 | 132 | 128 | 101 | 105 | - |
| Maximum | 234 | 234 | 229 |  | 234 | 219 | 226 | 233 | 210 | - |
| >140 (%) | 3976 (94.7) | 164 (97.6) | 136 (94.4) | 0.15 | 300 (96.2) | 421 (95.7) | 1023 (95.7) | 2139 (94.6) | 92 (81.4) | < 0.001 |
| DBP (mmHg) | 89.5 (13.3) | 92.2 (14.5) | 89.4 (13.9) | 0.086 | 90.9 (14.3) | 91.3 (13) | 89 (12.8) | 89.3 (13.4) | 88.5 (15.2) | 0.008 |
| Heart rate (bpm) | 77.5 (14.8) | 79.8 (16) | 76.3 (14.6) | 0.044 | 78.2 (15.4) | 79.8 (16) | 77.8 (14.3) | 76.9 (14.6) | 76.4 (15.8) | 0.003 |
| IS (%) | 3502 (83.5) | 128 (76.2) | 105 (72.9) | 0.51 | 233 (74.7) | 370 (84.1) | 896 (83.8) | 1897 (83.9) | 103 (91.2) | < 0.001 |
| ICH (%) | 646 (15.4) | 34 (20.2) | 33 (22.9) | 0.57 | 67 (21.5) | 65 (14.8) | 165 (15.4) | 340 (15.0) | 9 (8.0) | 0.007 |
| Alteplase (%) ‡ | 435 (10.4) | 57 (34.1) | 46 (32.2) | 0.71 | 103 (33.2) | 48 (10.9) | 112 (10.5) | 172 (7.6) | 0 (0.0) | < 0.001 |

† Stroke severity measured post-randomisation at hospital admission

‡ Percentage includes IS and ICH patients

AF: atrial fibrillation; bpm: beats per minute; DBP: diastolic blood pressure; DM: diabetes mellitus; ICH: intracerebral haemorrhage; IS: ischaemic stroke; IHD: ischaemic heart disease; NR: not recorded; SBP: systolic blood pressure; SSS: Scandinavian Stroke Scale

**Supplemental Figure I. Flow diagram of identification of included and excluded trials of NO donors.**

Excluded trials

N = 3

Inclusion criteria applied

Excluded N = 285

(Animal studies, reviews, search overlap, irrelevant endpoints)

Included trials with individual patient data for meta-analysis

N = 5

Identified trials

N = 8

Records for evaluation after electronic database search, hand search of the reference lists and the other sources according to search strategy.

N = 293

**Supplemental Figure II**. Effect of glyceryl trinitrate versus no glyceryl trinitrate on functional outcome (modified Rankin Scale) at 90 days in pre-defined subgroups of patients who were all randomised within 6 hours of stroke onset. Analyses are adjusted.


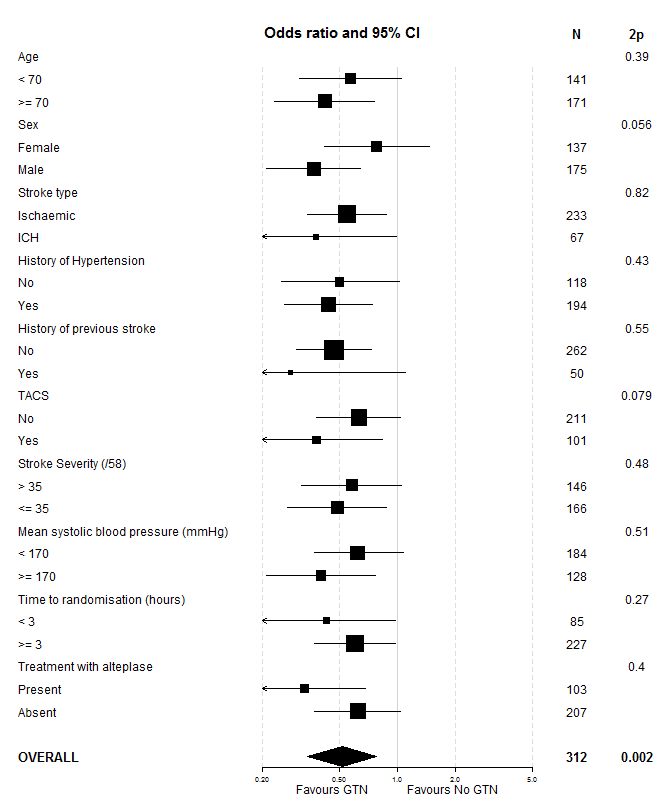


**Supplemental Figure III**. Distribution in day 90 modified Rankin Scale scores for glyceryl trinitrate versus no glyceryl trinitrate in 67 patients with intracerebral haemorrhage who were randomised within 6 hours of stroke onset. Common odds ratio 0.35 (95% confidence intervals 0.13-0.93, p=0.035).

**Supplemental Figure IV**. Distribution in day 90 modified Rankin Scale scores for glyceryl trinitrate versus no glyceryl trinitrate in 233 patients with ischaemic stroke who were randomised within 6 hours of stroke onset. Common odds ratio 0.55 (95% confidence intervals 0.34-0.89; p=0.014).

**Supplemental Figure V**. Distribution in modified Rankin Scale scores at day 90 for glyceryl trinitrate versus no glyceryl trinitrate in 98 patients with ischaemic stroke who were randomised within 6 hours of stroke onset and who received alteplase. Common odds ratio 0.32 (95% confidence intervals 0.15-0.69; p=0.004).

**Supplemental Figure VI**. Distribution in modified Rankin Scale scores at day 90 for glyceryl trinitrate versus no glyceryl trinitrate in 133 patients with ischaemic stroke who were randomised within 6 hours of stroke onset and who did not receive alteplase. Common odds ratio 0.80 (95% confidence intervals 0.42-1.51; p=0.49), proportional odds assumption test p=0.22; Mann-Whitney U test, p=0.092.

**Supplemental References**

1. Bath PM, Pathansali R, Iddenden R, Bath FJ. The effect of transdermal glyceryl trinitrate, a nitric oxide donor, on blood pressure and platelet function in acute stroke. *Cerebrovasc Dis*. 2001;11:265-272

2. Rashid P, Weaver C, Leonardi-Bee J, Bath F, Fletcher S, Bath P. The effects of transdermal glyceryl trinitrate, a nitric oxide donor, on blood pressure, cerebral and cardiac hemodynamics, and plasma nitric oxide levels in acute stroke. *J Stroke Cerebrovasc Dis*. 2003;12:143-151

3. Willmot M, Ghadami A, Whysall B, Clarke W, Wardlaw J, Bath PMW. Transdermal glyceryl trinitrate lowers blood pressure and maintains cerebral blood flow in recent stroke. *Hypertension*. 2006;47:1209-1215

4. Ankolekar S, Sare G, Geeganage C, Fuller M, Stokes L, Sprigg N, et al. Determining the feasibility of ambulance-based randomised controlled trials in patients with ultra-acute stroke: Study protocol for the "rapid intervention with gtn in hypertensive stroke trial" (right, isrctn66434824). *Stroke Res Treat*. 2012;2012:385753

5. Ankolekar S, Fuller M, Cross I, Renton C, Cox P, Sprigg N, et al. Feasibility of an ambulance-based stroke trial, and safety of glyceryl trinitrate in ultra-acute stroke: The rapid intervention with glyceryl trinitrate in hypertensive stroke trial (right, isrctn66434824). *Stroke*. 2013;44:3120-3128

6. Ankolekar S, Parry R, Sprigg N, Siriwardena AN, Bath PM. Views of paramedics on their role in an out-of-hospital ambulance-based trial in ultra-acute stroke: Qualitative data from the rapid intervention with glyceryl trinitrate in hypertensive stroke trial (right). *Ann Emerg Med*. 2014

7. The ENOS Trial Investigators. Glyceryl trinitrate vs. Control, and continuing vs. Stopping temporarily prior antihypertensive therapy, in acute stroke: Rationale and design of the efficacy of nitric oxide in stroke (enos) trial (isrctn99414122). *International Journal of Stroke*. 2006;1:245-249

8. Bath PM, Houlton A, Woodhouse L, Sprigg N, Wardlaw J, Pocock S. Statistical analysis plan for the 'efficacy of nitric oxide in stroke' (enos) trial. *Int J Stroke*. 2014;9:372-374

9. Bath PMW, Woodhouse L, Scutt P, Krishnan K, Wardlaw JM, Bereczki D, et al. Management of high blood pressure in acute stroke: Efficacy of nitric oxide in stroke (enos), a partial-factorial randomised controlled trial. *Lancet*. 2014;In Press

10. ENOS Investigators. Baseline characteristics of the 4011 patients recruited into the "efficacy of nitric oxide in stroke (enos) trial. *International Journal of Stroke*. 2014;9:711-720

11. Higgins JPT, Green S. *Cochrane handbook for systematic reviews of interventions* 2011.

**Supplemental Search criteria**

Medical Subject Headings (MeSH)

Administration, Transdermal; Arginine [*therapeutic use]; Brain Ischemia [*drug therapy]; Enzyme Inhibitors [*therapeutic use]; Isosorbide dinitrate; Nitric oxide [administration and dosage; adverse events]; Nitric Oxide Donors [*therapeutic use]; Nitric Oxide synthase [*antagonists and inhibitors]; Nitroglycerine; Nitrosoglutathione; [administration and dosage; adverse events]; stroke [*drug therapy]; vasodilator agents [adverse effects; *therapeutic use]; Randomized Controlled Trials as Topic

MeSH check words

Adult; child; humans

**MEDLINE search strategy**

1. blood pressure.tw.
2. hypertension.tw
3. acute/
4. stroke.tw.
5. or/1-4
6. and/1-4
7. 1-4.k.f.
8. 1-4.ti.
9. randomised controlled trial
10. controlled clinical trial
11. randomised
12. placebo
13. 1-4 and 9
14. 1-4 and 9.to.
15. 1-4 and 10.to.
16. 1-4 and 11.to.
17. 1-4 and 12.to.
18. ischaemic stroke.tw/ti.
19. haemorrhagic stroke.tw/ti.
20. intracerebral haemorrhage.tw./ti
21. blood pressure lowering/
22. 1-4 or 12
23. 1-4 or 13
24. 1-4 or 14
25. 1-4 and 15
26. cerebr
27. nitrate.tw.
28. glyceryl trinitrate/GTN.tw
29. nitric oxide donors.tw.
30. nitrate.tw.
31. nitroglycerin.tw.
32. trinitrate.tw.
33. dinitrate.tw.
34. mononitrate.tw.
35. nitroprusside.tw.
36. 1-4 and/or 27-35
37. 9-13 and/or 26-35

**Embase search strategy**

1. blood pressure.tw.
2. hypertension.tw
3. acute/
4. stroke.tw.
5. or/1-4
6. and/1-4
7. 1-4.k.f.
8. 1-4.ti.
9. randomised controlled trial
10. controlled clinical trial
11. randomised
12. placebo
13. 1-4 and 9
14. 1-4 and 9.to.
15. 1-4 and 10.to.
16. 1-4 and 11.to.
17. 1-4 and 12.to.
18. ischaemic stroke.tw/ti.
19. haemorrhagic stroke.tw/ti.
20. intracerebral haemorrhage.tw./ti
21. blood pressure lowering/
22. 1-4 or 12
23. 1-4 or 13
24. 1-4 or 14
25. 1-4 and 15
26. cerebr
27. nitrate.tw.
28. glyceryl trinitrate/GTN.tw
29. nitric oxide donors.tw.
30. nitrate.tw.
31. nitroglycerin.tw.
32. trinitrate.tw.
33. dinitrate.tw.
34. mononitrate.tw.
35. nitroprusside.tw.
36. 1-4 and/or 27-35
37. 9-13 and/or 26-35

**Science Citation Index search strategy**

1. blood pressure.TI.
2. hypertension.TI
3. acute/
4. stroke.TS.
5. OR/1-4
6. AND/1-4
7. 1-4.TI.
8. 1-4.TI.
9. trials
10. 1-4 and 9
11. 1-4 AND 9.ti.
12. ischaemic stroke/TI/TS.
13. haemorrhagic stroke.TI/TS.
14. intracerebral haemorrhage.TI./TS.
15. blood pressure lowering/
16. cerebr
17. 1-4 OR 12
18. 1-4 OR 13
19. 1-4 OR 14
20. 1-4 OR 15
21. 1-4 OR 16
22. nitrate.TI./TS
23. glyceryl trinitrate/GTN/TI/TS
24. nitric oxide donors.TI./TS.
25. mononitrate.TI/TS
26. nitroprusside.tw.
27. 1-4 AND/OR 22-26
28. autoregulation
